# Evidence for long-acting methylprednisolone acetate in dogs: A critically appraised topic on efficacy, safety, and clinical applications across administration routes

**DOI:** 10.14202/vetworld.2026.3401-3414

**Published:** 2026-08-08

**Authors:** Jevgenija Kondratjeva, Aija Ilgaza

**Affiliations:** 1 Preclinical Institution, Faculty of Veterinary Medicine, Latvia University of Life Sciences and Technologies, Jelgava, LV-3004, Latvia.

**Keywords:** adverse effects, canine medicine, depot glucocorticoids, evidence-based medicine, intervertebral disc disease, methylprednisolone acetate, route of administration, safety evaluation

## Abstract

**Background and Aim:**

Long-acting methylprednisolone acetate (MPA) is widely used in canine medicine for its prolonged glucocorticoid activity. However, uncertainty remains regarding its disease-specific efficacy, safety profile, and overall benefit-risk balance across different routes of administration. This critically appraised topic (CAT) aimed to systematically evaluate and synthesize the available evidence on the efficacy, safety, and clinical applications of depot MPA in dogs, with emphasis on clinically meaningful outcomes and evidence-based recommendations for veterinary practice.

**Materials and Methods:**

A structured literature search was performed in PubMed, CAB Abstracts, and Scopus from database inception to May 17, 2026. Eligible studies evaluated depot MPA administered through any route in dogs and reported clinical efficacy, safety outcomes, pharmacokinetics, endocrine responses, or clinicopathological changes. Study quality was assessed using design-specific risk-of-bias tools, including Cochrane RoB 2, ROBINS-I, and SYRCLE's risk-of-bias tool. Data were synthesized narratively todue of substantial heterogeneity in study designs, routes of administration, and outcome measures.

**Results:**

Fourteen studies involving more than 200 dogs were included, comprising one blinded randomized controlled trial, two clinical cohort studies, one case series, and ten pharmacokinetic, physiological, safety, or experimental investigations. The strongest evidence supported epidural MPA administered during surgery for thoracolumbar intervertebral disc disease (IVDD), significantly reducing the median time to ambulation from 7 to 3 days. Evidence for degenerative lumbosacral stenosis demonstrated favorable short-term clinical improvement but frequent relapse during long-term follow-up. Perineural, paravertebral, intra-articular, and musculoskeletal applications were supported only by low-level evidence. Pharmacokinetic and physiological studies consistently demonstrated prolonged systemic exposure, hypothalamic-pituitary-adrenal axis suppression, and measurable clinicopathological changes after depot administration. However, controlled evidence supporting routine systemic intramuscular MPA for naturally occurring inflammatory or immune-mediated diseases was lacking. Adverse event reporting was inconsistent, with prolonged endocrine suppression, systemic glucocorticoid effects, and occasional serious complications reported.

**Conclusion:**

Current evidence indicates that the clinical utility of depot MPA in dogs is highly route dependent. Moderate-quality evidence supports epidural administration for selected cases of thoracolumbar IVDD, whereas evidence for other regional applications remains limited. Routine systemic intramuscular depot MPA cannot currently be recommended as an evidence-based first-line treatment because disease-specific comparative trials are lacking. This CAT provides the first comprehensive evidence synthesis evaluating depot MPA across multiple administration routes in dogs, identifies important knowledge gaps, and highlights priorities for future controlled clinical studies to optimize therapeutic decision-making.

## INTRODUCTION

Long-acting depot glucocorticoids are widely used in veterinary clinical practice because they simplify treatment protocols, improve owner compliance, and reduce the need for repeated drug administration. Methylprednisolone acetate (MPA) is a particulate, water-insoluble glucocorticoid formulation that provides prolonged systemic exposure compared with soluble methylprednisolone preparations in dogs [1]. Consistent with its pharmacokinetic profile, controlled physiologic studies have demonstrated sustained systemic biologic effects following MPA administration, including prolonged suppression of the hypothalamic-pituitary-adrenal (HPA) axis and measurable clinicopathologic alterations [2–5]. Although these prolonged effects may provide therapeutic advantages in selected clinical situations, they also limit the ability to rapidly reduce the dose or discontinue treatment if adverse events (AE) occur. In routine clinical practice, systemic intramuscular (IM) depot MPA is commonly administered at 1–2 mg/kg, with repeat injections determined by disease severity and clinical response. The clinical effect typically persists for approximately 3 weeks but may range from 1 to >4 weeks. Product labeling also describes an average IM dose of 20 mg per dog, with doses ranging from 2 mg in miniature breeds to 120 mg in giant breeds or dogs with severe disease. Furthermore, MPA suspensions (Depo-Medrone V®, Pfizer; Depo-Medrol®, Zoetis) are licensed for veterinary use in some jurisdictions [6–8]. Current product information indicates their use for allergic and inflammatory dermatoses, musculoskeletal disorders, otic and ophthalmic inflammatory diseases, arthritis, and other corticosteroid-responsive conditions in dogs, although approved indications and label wording differ among countries and formulations [6–8].

Species-specific differences should also be considered when interpreting physiologic studies and extrapolating glucocorticoid pharmacology. Compared with dogs, cats possess approximately half as many glucocorticoid receptors and lower receptor affinity in the liver and skin, which may contribute to their relative glucocorticoid resistance and limit direct interspecies extrapolation [9]. Likewise, glucocorticoid administration in horses is associated with unique safety concerns, including altered glucose metabolism, reduced insulin sensitivity, and an increased risk of laminitis. Moreover, intra-articular administration of MPA has been shown to suppress endogenous hydrocortisone secretion despite local administration [10–12]. Collectively, these findings demonstrate that glucocorticoid potency, HPA axis suppression, pharmacologic responses, and adverse effect profiles differ among species and emphasize the importance of relying on canine-specific evidence when evaluating the benefit-risk profile of depot MPA in dogs.

Despite its widespread clinical use, uncertainty remains regarding whether the disease-specific efficacy and overall benefit-risk profile of depot MPA have been adequately evaluated in naturally occurring inflammatory and immune-mediated diseases in dogs, particularly when compared with short-acting oral glucocorticoids that permit dose titration, tapering, and rapid discontinuation. Currently available evidence is primarily focused on regional or local applications, such as epidural and perineural administration, rather than on routine systemic IM use [13–16]. Evidence supporting intra-articular administration in naturally occurring disease is gradually emerging. Comparative clinical studies have evaluated intra-articular MPA against other glucocorticoids in dogs with osteoarthritis (OA) [17], whereas recent reviews continue to emphasize that, despite widespread clinical use and product licensing, high-quality clinical trials in naturally occurring OA remain limited [18]. Furthermore, recent studies have demonstrated that recovery of HPA axis function following intra-articular glucocorticoid administration may require up to 7 weeks, highlighting the importance of endocrine monitoring and prolonged safety assessment when long-acting glucocorticoid formulations are used [19].

Although depot MPA has been used in canine medicine for several decades, the available evidence remains fragmented and heterogeneous with respect to route of administration, study design, patient population, and reported outcomes. Most published investigations have focused on pharmacokinetic characteristics, physiologic responses, endocrine suppression, or localized therapeutic applications, whereas robust randomized controlled trials (RCTs) evaluating disease-specific clinical efficacy in naturally occurring canine disorders are scarce. Furthermore, direct comparisons between systemic IM depot MPA and titratable oral glucocorticoids are largely unavailable, making it difficult to determine their relative efficacy, safety, and clinical utility. Existing studies also vary considerably in follow-up duration, adverse event reporting, and outcome assessment, limiting evidence-based clinical decision-making. Consequently, there is currently no comprehensive critical synthesis that integrates the available evidence across all routes of administration and evaluates both therapeutic effectiveness and the overall benefit-risk profile of depot MPA in dogs.

Therefore, this critically appraised topic (CAT) aimed to systematically identify, critically appraise, and synthesize the available evidence regarding the efficacy, safety, pharmacologic effects, and clinical applications of depot MPA in dogs across all reported routes of administration, including systemic IM, epidural/neuraxial, intra-articular, and perineural techniques. Particular emphasis was placed on clinically meaningful patient outcomes, adverse effects, quality of evidence, and the overall benefit-risk profile of depot MPA compared with current therapeutic alternatives. Furthermore, this CAT sought to identify important knowledge gaps and provide evidence-based recommendations to support rational clinical decision-making and guide future controlled clinical research in canine medicine.

## MATERIALS AND METHODS

### Ethical approval

Ethical approval was not required because this study was a critically appraised topic based solely on previously published data and did not involve animals, human participants, or the collection of new experimental data. 

### Study period and location

The literature search, data extraction, quality appraisal, data analysis, and interpretation were conducted between February and May 2026 at the Latvia University of Life Sciences and Technologies, Jelgava, Latvia. 

### Study design

This study was conducted as a CAT using a structured evidence-based approach to identify, critically appraise, and synthesize the available literature regarding the efficacy, safety, pharmacokinetics (PK), physiologic effects, and clinical applications of depot MPA in dogs. The review followed a predefined Population, Intervention, Comparison, and Outcome (PICO) framework comprising systematic literature searching, study selection, quality assessment, and narrative synthesis of evidence.

### Clinical scenario

A clinician is considering methylprednisolone therapy for a dog with an inflammatory condition. The owner requests a depot injection because it would be more convenient than an oral treatment regimen. Therefore, the clinician seeks to determine whether MPA has been adequately evaluated in dogs with naturally occurring diseases with respect to clinically meaningful efficacy outcomes and its overall benefit-risk profile.

### Refining the question

A PICO question was formulated:

In dogs, does depot MPA improve clinically meaningful outcomes?

1. **P (population):** Dogs with diseases for which glucocorticoid therapy is indicated.

2. I (intervention): Depot MPA.

3. **C (comparison):** Placebo, untreated controls, or short-acting oral glucocorticoids (prednisolone or prednisone).

4. **O (outcome):** Clinically meaningful outcomes and evidence informing the overall benefit-risk profile.

The preferred study designs were RCTs and controlled clinical trials involving naturally occurring canine diseases.

### Search strategy

A comprehensive literature search was performed using PubMed, CAB Abstracts, and Scopus from database inception to May 17, 2026. The following search strategy was applied:

((dog OR dogs OR canine) AND ("methylprednisolone acetate"))

**Figure 1:**
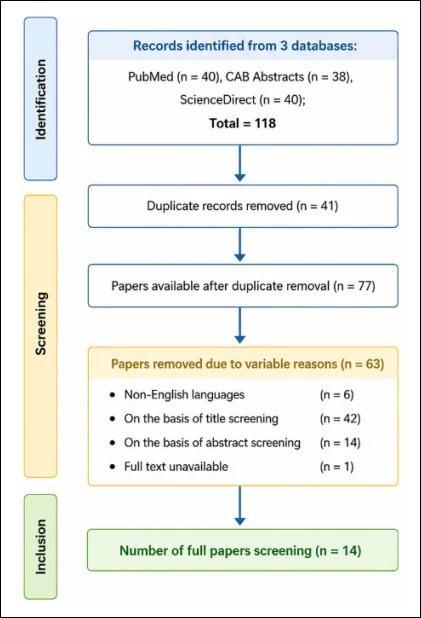
Search strategy and study selection process flowchart.

The review was conducted in accordance with the updated Preferred Reporting Items for Systematic Reviews and Meta-Analyses (PRISMA) guidelines. The literature search and study selection process are summarized in Figure 1.

### Eligibility criteria

Studies were considered eligible if they evaluated depot MPA administered to dogs by any route, including systemic IM, epidural/neuraxial, intra-articular, perineural/paravertebral, subconjunctival, or other clinically relevant routes, and reported clinically meaningful efficacy outcomes or data relevant to the benefit-risk profile. Eligible outcomes included efficacy or effectiveness, AE, safety signals, PK, endocrine or physiologic effects (e.g., HPA axis suppression), and clinicopathologic biomarker changes.

For this CAT, clinically meaningful outcomes were defined as patient-important endpoints reflecting functional improvement, therapeutic success, or safety. These outcomes included time to ambulation, return to independent walking, pain reduction, improvement in functional performance or owner-reported outcomes, recurrence or relapse, the need for additional treatment, and AE. Because of the considerable heterogeneity among the included studies, no formal minimal clinically important difference threshold was predefined. Instead, clinical relevance was interpreted according to the primary outcomes reported by the original study authors.

Studies were excluded if they involved non-canine species, did not evaluate MPA, did not use a depot MPA formulation, were conference abstracts lacking sufficient methodological or outcome information, or used MPA solely as an experimental immunosuppressive or provocation agent without extractable outcomes relevant to the objectives of this CAT.

### Study selection and screening process

All retrieved records were collated, and duplicate records were removed before screening. Title and abstract screenings were performed independently by two reviewers. Potentially eligible articles subsequently underwent independent full-text assessment by both reviewers. Reasons for study exclusion are summarized in Figure 1.

Non-English publications were also assessed during the screening process; however, they did not provide clinically meaningful outcomes or extractable data relevant to the predefined PICO question and were therefore excluded from the final evidence synthesis.

The PubMed search retrieved 40 records. Following screening and eligibility assessment, 13 studies met the inclusion criteria and were retained. Searches of CAB Abstracts and Scopus identified one additional eligible study that was not indexed in PubMed. Consequently, the final evidence set comprised 14 studies (Figure 1).

Inter-reviewer agreement was formally assessed during title and abstract screening. Among the 77 records screened after duplicate removal, both reviewers agreed on the inclusion of 12 studies and the exclusion of 58 studies, whereas disagreement occurred for seven records. This corresponded to an overall agreement of 90.9% and substantial inter-reviewer reliability (Cohen's κ = 0.72). All disagreements were resolved through discussion until consensus was reached.

### Quality appraisal

Given the heterogeneity in routes of administration and study designs, methodological quality was assessed using design-specific risk of bias tools. RCTs were evaluated using the Cochrane Risk of Bias 2 (RoB 2) tool, non-randomized clinical studies were assessed using the Risk of Bias in Non-Randomized Studies of Interventions (ROBINS-I) tool, and experimental animal studies were evaluated using the SYRCLE risk of bias tool.

The RCT conducted by Natsios *et al.* [16] demonstrated a low risk of bias for the randomization process, deviations from intended interventions, missing outcome data, and selection of the reported results. Outcome assessment was judged to have some concerns because the postoperative neurologic evaluation inherently involved clinical judgment, although blinding was maintained throughout the study. Overall, the trial was considered to have a low risk of bias.

The retrospective DLSS study conducted by Janssens *et al.* [13] demonstrated a serious overall risk of bias according to the ROBINS-I assessment. The principal concerns were related to confounding and selection bias because treatment allocation was not randomized and case selection depended on clinical decision-making. Outcome assessment was also considered to be at serious risk of bias because clinical improvement was assessed predominantly using owner-reported outcomes, with variable follow-up periods. Missing data were judged to have a moderate risk of bias, whereas selective reporting was considered to have a low risk.

The prospective DLSS study conducted by Gomes *et al.* [14] demonstrated a moderate-to-serious overall risk of bias. Confounding remained an important concern because no concurrent control group was included and the influence of co-interventions could not be completely excluded. Selection bias was considered moderate, whereas outcome assessment carried a moderate risk because clinical follow-up and recurrence assessments were not fully standardized. Missing outcome data were judged to have a low-to-moderate risk of bias, and selective reporting was considered to have a low risk.

PK, physiologic, and biomarker studies without clinical efficacy endpoints were appraised using predefined internal validity criteria, including the clarity of the dose and route of administration, the validity of outcome measurements, the completeness of follow-up, and the risk of selective reporting. Owing to substantial methodological heterogeneity among the included studies, the evidence was synthesized narratively by route of administration and clinical indication. Patient-important outcomes were prioritized when formulating efficacy conclusions, whereas physiologic and PK findings were used to provide additional context for the overall benefit-risk profile.

## RESULTS

### Quality of the evidence

The overall quality of evidence was heterogeneous, with substantial route-specific differences in study design, outcome assessment, sample size, follow-up duration, and certainty of evidence. The methodological quality and principal findings of the included studies are summarized in Table 1.

The strongest disease-specific evidence was derived from a prospective randomized, blinded controlled trial evaluating epidural MPA in dogs undergoing surgery for thoracolumbar IVDD. This study used time to ambulation as a clinically meaningful functional endpoint and was judged to have a low overall risk of bias [16]. Evidence supporting epidural MPA for DLSS was less certain because it was based on non-randomized studies that relied in part on subjective or owner-reported outcomes [13, 14]. Evidence for other local applications, including perineural, paravertebral, and musculoskeletal administration, was limited to small case series or retrospective studies with heterogeneous outcome assessments [15, 20].

**Table 1:** Quality appraisal of studies evaluating methylprednisolone acetate in dogs.

**Study**	**Indication/** **population**	**Route**	**Study design**	**Sample size and dose**	**Primary endpoint**	**Main finding**	**Reported AE/safety signals**	**Risk of bias**
Natsios, 2025 [16]	Dogs with non-ambulatory thoracolumbar IVDD undergoing surgery	Epidural, local intraoperative administration	Blinded RCT	n = 41: MPA, n = 18; control, n = 23; 1 mg/kg	Time to ambulation	Median time to ambulation was 3 days (range: 1–8 days) with MPA and 7 days (range: 1–17 days) in controls, representing an approximately 4-day faster median recovery (p = 0.01)	Discospondylitis and abscess formation in 1 of 18 treated dogs	Low risk of bias because of randomization, blinding, and an objective primary outcome; limited power to detect AE because of the small sample size
Janssens, 2009 [13]	Dogs with DLSS	Epidural	Retrospective clinical study	n = 38; 1 mg/kg; minimum volume, 0.5 mL; protocol included at least three injections	Owner-reported clinical response	Improvement in 30 of 38 dogs (79%); complete resolution in 20 of 38 dogs (53%); significant improvements in lameness, rising, and jumping (p < 0.001 for all outcomes)	AE were not systematically quantified	Serious risk of bias because of the retrospective design, absence of a control group, subjective owner-reported outcomes, variable follow-up, and potential recall bias
Gomes, 2020 [14]	Dogs with DLSS	Epidural	Prospective cohort study	n = 32; 1 mg/kg; minimum volume, 0.5 mL	Clinical outcome and owner-reported QoL	Improvement in 27 of 32 dogs (84.4%); relapse in 17 of 22 initial responders (77.2%); sustained response in 5 of 32 dogs (15.6%); mean follow-up, 9.4 months	Systemic glucocorticoid-related signs, including polyphagia and polydipsia, were specifically assessed, but rates were not fully quantified	Moderate-to-serious risk of bias because of the absence of randomization and a concurrent control group and the inclusion of subjective outcome components
Wolf, 2021 [15]	Dogs with refractory cervical pain	Perineural/paravertebral	Case series	n = 4; 1 mg/kg	Clinical response	Clinical improvement was reported in all dogs	NR	Serious risk of bias because of the very small sample size, uncontrolled design, and absence of standardized outcome assessment
Stobie, 1995 [20]	Dogs with bicipital tenosynovitis	Local administration within the treatment pathway	Retrospective case series	n = 29 affected shoulder joints; MPA administered to 21 joints	Clinical outcome	Clinical outcomes were described, but the specific effect of MPA could not be isolated	NR	Serious risk of bias because of the retrospective design, multiple co-interventions, and absence of an MPA-specific comparative analysis
Liotta, 2016 [18]	Healthy dogs	Spinal-region injection	Prospective feasibility and safety study	n = 15; 1 mg/kg; successful injection in 14 of 15 dogs	Technical feasibility and safety	Correct placement was achieved in 14 of 15 dogs	Mild transient hyperthermia, reflex changes, and one vascular puncture; no major complications	Moderate risk of bias because of the small sample size and use of healthy dogs rather than dogs with naturally occurring disease
Pelletier, 1994 [22]	Dogs with experimentally induced OA	Intra-articular	Controlled experimental study	n = 15: MPA, n = 8; control, n = 7; 1 mg/kg administered twice at 4-week intervals	Histologic lesion score	MPA reduced OA lesion severity compared with the control treatment	NR	Moderate risk of bias because the study used an experimental OA model rather than naturally occurring disease
Spencer, 1980 [2]	Healthy dogs	IM	Controlled physiologic study	n = 25, comprising five groups of five dogs; MPA group, n = 5; 2.5 mg/kg administered nine times at 1-week intervals	ACTH response and adrenal weight	Significant suppression of ACTH response and adrenal atrophy were observed (p < 0.05)	No structured clinical AE reporting	Moderate risk of bias because of small group sizes, lack of blinding, and reliance on surrogate physiologic endpoints
Kemppainen, 1981 [3]	Healthy dogs	IM	Controlled physiologic study	n = 12; single dose of 2.5 mg/kg	ACTH stimulation response	HPA axis suppression persisted for at least 5 weeks (p < 0.02–0.001)	Prolonged HPA axis suppression indicated an extended biologic risk window; clinical AE rates were not quantified	Moderate risk of bias because of the small sample size and reliance on surrogate endocrine outcomes rather than clinical endpoints
Regnier, 1982 [5]	Healthy dogs	Subconjunctival	Controlled physiologic study	n = 6; 10 mg administered every 21 days	Hematologic and biochemical changes	Significant biochemical alterations were detected	NR	Moderate risk of bias because of the very small sample size and reliance on surrogate biochemical outcomes
Braun, 1981 [4]	Healthy dogs	IM	Prospective clinicopathologic study	n = 24: control, n = 6; MPA, n = 18; 4 mg/kg	Hematologic and biochemical changes	Measurable systemic clinicopathologic changes occurred after MPA administration	Weekly physical examinations, complete blood counts, and serum biochemical analyses were performed; systematic clinical AE rates were NR	Moderate risk of bias because of small group sizes, lack of blinding, and the limited emphasis on clinical AE
Martínez-Subiela, 2004 [23]	Healthy dogs	SC	Prospective comparative study	n = 21; MPA group, n = 7; 1.1 mg/kg	APP concentrations	Significant changes in APP concentrations occurred after MPA administration	AE reporting was not a primary study objective	Moderate risk of bias because of the small sample size and reliance on surrogate biomarker outcomes
Toutain, 1986 [1]	Healthy dogs	IM	Prospective PK study	n = 5; 4 mg/kg	Plasma PK parameters	Depot MPA produced prolonged systemic exposure	PK-only study; clinical AE were not evaluated	Moderate risk of bias because of the very small sample size and absence of clinical efficacy outcomes
Rijsdijk, 2012 [24]	Dogs in a preclinical neuraxial model	Intrathecal	Prospective safety and PK study	n = 17: vehicle, n = 4; MPA 20 mg/mL, n = 7; MPA 80 mg/mL, n = 6; four administrations at 7-day intervals	Safety and PK	Intrathecal MPA caused inflammatory lesions, with greater long-term inflammation at higher doses (long-term p = 0.014; acute p = 0.167)	Brief motor block, dose-dependent CSF abnormalities, increased ALP activity in one dog, leukopenia in two dogs in the high-dose group, and dose-dependent long-term inflammation	Moderate risk of bias despite randomization and blinded histopathologic assessment because this was a preclinical safety model rather than a clinical efficacy study

ACTH = Adrenocorticotropic hormone; AE = Adverse event(s); ALP = Alkaline phosphatase; APP = Acute-phase protein(s); CSF = Cerebrospinal fluid; DLSS = Degenerative lumbosacral stenosis; HPA axis = Hypothalamic-pituitary-adrenal axis; IM = Intramuscular; IVDD = Intervertebral disc disease; MPA = Methylprednisolone acetate; NR = Not reported; OA = Osteoarthritis; PK = Pharmacokinetics; QoL = Quality of life; RCT = Randomized controlled trial; SC = Subcutaneous.

The 14 included studies comprised one blinded RCT of epidural MPA in dogs with IVDD, two clinical studies of epidural MPA in dogs with DLSS, one perineural case series, and several PK, physiologic, biomarker, feasibility, and safety investigations. The evidence base was therefore highly heterogeneous with respect to route of administration, study population, comparator, outcome type, and clinical relevance.

PK, physiologic, and biomarker studies consistently demonstrated prolonged systemic biologic activity after depot MPA administration but did not establish disease-specific clinical efficacy in naturally occurring canine diseases [1–5, 19, 23]. Nevertheless, these studies contributed important benefit-risk information by demonstrating prolonged HPA axis suppression and measurable systemic clinicopathologic changes after exposure.

The quality of AE reporting varied substantially across studies. Most investigations had small sample sizes, lacked standardized definitions of AE, and did not use systematic or sufficiently long follow-up periods. Consequently, safety signals were frequently inferred from endocrine, physiologic, or biomarker changes rather than from prospectively collected clinical AE rates.

Quantitative synthesis was not feasible because the included studies differed substantially in routes of administration, clinical populations, study designs, outcome definitions, and follow-up periods. Time to ambulation was reported in only one blinded RCT involving dogs with IVDD, whereas the DLSS studies used non-randomized designs and different clinical outcome frameworks. In addition, the PK and physiologic studies primarily reported surrogate outcomes rather than patient-important clinical endpoints.

### Clinical applicability of the evidence

The clinical applicability of depot MPA varied considerably according to the route of administration, indication, methodological quality, and duration of follow-up. The clinical implications and major evidence gaps are summarized in Table 2.

### Epidural administration for thoracolumbar IVDD

The highest-quality evidence for a clinical benefit of depot MPA was route-specific. In non-ambulatory dogs undergoing decompressive surgery for thoracolumbar IVDD, intraoperative epidural MPA at 1 mg/kg significantly reduced the median time to ambulation from 7 days (range: 1–17 days) in the control group to 3 days (range: 1–8 days) in the treatment group, corresponding to an approximately 4-day faster median recovery [16].

**Table 2:** Clinical applicability of methylprednisolone acetate in dogs.

**Evidence cluster**	**Available evidence**	**Key study/studies**	**Dose(s) used in included studies**	**Comparator**	**Reported AE/safety signals**	**Follow-up**	**Major evidence gaps**	**Clinical implication**
Highest-quality clinical evidence	One blinded RCT demonstrated improved functional recovery after epidural MPA in dogs with IVDD [16]	Natsios, 2025 [16]	Epidural, intraoperative, 1 mg/kg	Control group without epidural MPA	Discospondylitis and abscess formation in 1 of 18 treated dogs; surgical site infection, delayed wound healing, worsening neurologic deficits, and cystitis were monitored	Short-term postoperative follow-up until ambulation	Independent replication, comparison with oral glucocorticoids, adequately powered safety studies	Strongest evidence supports this specific local intraoperative application
Epidural administration for DLSS	Prospective and retrospective clinical studies demonstrated improvement, but recurrence was common [13, 14]	Janssens, 2009 [13]; Gomes, 2020 [14]	Epidural, 1 mg/kg; minimum injection volume, 0.5 mL; MPA 40 mg/mL	No control group	Transient PU/PD, temporary worsening of pain, and delayed recovery from sedation were reported in Janssens *et al.* [13]; transient systemic glucocorticoid-related signs (polyphagia and PU/PD) were reported in one dog in Gomes *et al.* [14]	Owner follow-up, 5–66 months [13]; mean follow-up, 9.4 months [14]	Placebo-controlled RCTs, standardized outcome measures, long-term durability	Suggestive clinical benefit but insufficient evidence for definitive recommendations
Perineural/paravertebral administration	Small uncontrolled case series only [15]	Wolf, 2021 [15]	Perineural/paravertebral, 1 mg/kg	None	No major complications; transient post-injection pain in one dog	Short-term	Controlled trials, objective pain assessment, systematic safety evaluation	Evidence remains insufficient to support routine evidence-based use
Musculoskeletal and intra-articular administration	Experimental OA model and retrospective clinical pathway studies [20, 22]	Pelletier, 1994 [22]; Stobie, 1995 [20]	Variable or NR	Experimental controls only in OA model; none in clinical study	No complications associated with medical treatment were reported by Stobie *et al.* [20]; no systematic clinical AE reported in the OA model [22]	No meaningful long-term clinical follow-up	RCTs in naturally occurring OA and bicipital tenosynovitis	Experimental and retrospective evidence suggests biologic plausibility but not clinical proof
Systemic IM administration	Evidence limited to PK, endocrine, physiologic, and clinicopathologic studies [1–5, 23]	Toutain, 1986 [1]; Spencer, 1980 [2]; Kemppainen, 1981 [3]; Braun, 1981 [4]; Regnier, 1982 [5]; Martínez-Subiela, 2004 [23]	IM, 2.5–4 mg/kg; SC, 1.1 mg/kg	Baseline or untreated controls only	Prolonged HPA axis suppression lasting several weeks and systemic clinicopathologic and biomarker alterations	Physiologic follow-up demonstrated suppression lasting at least 5 weeks	Controlled clinical trials for naturally occurring diseases; direct comparison with oral glucocorticoids	Current evidence supports prolonged biologic activity and associated risks but not disease-specific clinical efficacy
Route feasibility and safety	Technical feasibility studies and preclinical safety investigations [18, 24]	Liotta, 2016 [18]; Rijsdijk, 2012 [24]	CT-guided spinal injection, 1 mg/kg; repeated intrathecal administration in dose groups	No comparator in feasibility study; vehicle control in preclinical study	Mild transient hyperthermia and transient neurologic reflex changes after CT-guided injection; dose-dependent meningeal inflammation, CSF abnormalities, leukopenia, increased body weight, and brief motor block after intrathecal administration	Short-term feasibility assessment and longer-term preclinical safety evaluation	Large prospective clinical safety studies across administration routes	Supports procedural feasibility and highlights route-specific safety considerations rather than clinical efficacy

AE = Adverse event(s); CSF = Cerebrospinal fluid; CT = Computed tomography; DLSS = Degenerative lumbosacral stenosis; HPA axis = Hypothalamic-pituitary-adrenal axis; IM = Intramuscular; IVDD = Intervertebral disc disease; MPA = Methylprednisolone acetate; NR = Not reported; OA = Osteoarthritis; PK = Pharmacokinetics; PU/PD = Polyuria/polydipsia; RCT = Randomized controlled trial; SC = Subcutaneous.

One of the 18 dogs treated with MPA developed discospondylitis and abscess formation. However, the trial sample size was insufficient to reliably detect uncommon or delayed AE [16]. In addition, long-term follow-up beyond 6 months was not reported, limiting conclusions regarding recurrence, delayed complications, or sustained neurologic benefit.

### Epidural administration for DLSS

Evidence supporting epidural MPA for DLSS was derived from one prospective cohort and one retrospective clinical study [13, 14]. In the prospective cohort, 27 of 32 dogs (84.4%) improved after epidural injection. However, relapse occurred in 17 of 22 initial responders (77.2%) within 6 months, including 15 of 17 relapses within the first 2 months. A sustained favorable response was documented in only 5 of 32 dogs (15.6%) during a mean follow-up period of 9.4 months [14].

In the retrospective study, which included owner follow-up ranging from 5 to 66 months, 30 of 38 owners (79%) reported clinical improvement, and 20 of 38 (53%) reported complete resolution of clinical signs. Significant improvements were reported for lameness, difficulty rising, and jumping ability (p < 0.001) [13]. Nevertheless, both studies lacked placebo or untreated control groups and relied in part on subjective or owner-reported outcomes, increasing the risk of selection bias, attrition bias, and outcome-measurement bias.

Differences in diagnostic methods further limited comparison between the DLSS studies. In the retrospective study by Janssens *et al.* [13], diagnoses were not consistently confirmed with advanced imaging such as computed tomography (CT) or magnetic resonance imaging, and case inclusion was based in part on clinical findings and conventional radiography. This approach increased the possibility of case misclassification and reduced applicability to contemporary clinical practice, in which advanced imaging is generally used to confirm DLSS and guide treatment planning. Gomes *et al.* [14] used more standardized case selection; however, frequent relapse continued to limit confidence in long-term treatment durability.

### Perineural and paravertebral administration

Perineural or paravertebral depot MPA at 1 mg/kg for refractory cervical pain was evaluated only in a small uncontrolled case series involving four dogs [15]. Clinical improvement was reported in all dogs, but the absence of a comparator, objective standardized pain assessment, systematic AE monitoring, and long-term follow-up substantially limited interpretation. Therefore, the certainty of evidence supporting this application remains very low.

### Musculoskeletal and intra-articular administration

In dogs with chronic bicipital tenosynovitis, 21 of 29 affected shoulder joints received local MPA as part of a multimodal medical management protocol [20]. Outcomes were reported retrospectively, and several dogs received additional interventions, including surgery. These co-interventions prevented reliable attribution of the observed clinical outcomes specifically to MPA, and no controlled comparative trials were identified.

Experimental intra-articular MPA reduced osteoarthritic lesions in a surgically induced canine OA model [22]. However, these findings were obtained from experimentally induced disease and cannot be directly extrapolated to naturally occurring OA, routine clinical treatment, or systemic IM depot administration.

### Systemic IM administration

Evidence concerning systemic IM depot MPA was dominated by PK, endocrine, physiologic, and clinicopathologic studies. PK findings indicated prolonged methylprednisolone exposure compared with that of soluble formulations [1]. Endocrine studies showed sustained HPA axis suppression lasting several weeks after a single IM dose [2, 3, 5], whereas clinicopathologic studies documented measurable systemic biochemical and acute-phase protein changes after IM or SC administration [4, 23].

These findings confirm the prolonged biologic activity and systemic exposure associated with depot MPA but do not demonstrate disease-specific clinical efficacy. No controlled trials were identified that evaluated systemic IM depot MPA for common naturally occurring indications such as allergic dermatitis, immune-mediated polyarthritis, immune-mediated hemolytic anemia, or inflammatory bowel disease. The only related evidence was an early descriptive report concerning injectable MPA for canine allergy [25]. Therefore, contemporary evidence supporting the use of systemic IM depot MPA for allergic or immune-mediated diseases in dogs remains extremely limited.

### Procedural feasibility and route-specific safety

CT-guided spinal injections, including epidural and facet approaches, were technically feasible in healthy dogs receiving MPA at 1 mg/kg. Reported findings included mild transient hyperthermia and minor neurologic reflex changes [21]. However, evidence from healthy dogs provides limited information regarding clinical efficacy or safety in diseased populations.

In contrast, intrathecal administration was associated with dose-dependent inflammatory lesions, cerebrospinal fluid abnormalities, motor deficits, and leukopenia at higher doses [24]. These findings emphasize that safety cannot be generalized across routes of administration and warrant particular caution regarding direct intrathecal exposure.

### Duration of follow-up and evidence gaps

Long-term outcome data exceeding 6 months were limited across most evidence clusters. The retrospective DLSS study included owner follow-up ranging from 5 to 66 months [13], whereas relapse within 6 months was frequent in the prospective DLSS cohort [14]. No comparable long-term durability data were available for the epidural IVDD RCT or the perineural case series [15, 16].

No direct comparative trials of systemic IM depot MPA versus short-acting oral glucocorticoids were identified. This represents a major evidence gap because oral glucocorticoids permit dose adjustment, tapering, and prompt discontinuation when AE develop or the diagnosis changes. The absence of head-to-head studies also prevents reliable comparison of efficacy, treatment flexibility, cumulative glucocorticoid exposure, and overall benefit-risk between depot and oral treatment strategies.

## DISCUSSION

### Overall evidence for clinical efficacy

Based on the available evidence, MPA can be regarded as a pharmacologically long-acting glucocorticoid in dogs. This conclusion is supported by PK studies demonstrating prolonged systemic exposure and physiologic studies showing sustained biologic activity, including prolonged HPA axis suppression following administration [2, 3]. However, evidence supporting disease-specific clinical efficacy in naturally occurring canine diseases remains limited and is largely restricted to specific routes of administration. The strongest evidence supports intraoperative epidural MPA in dogs undergoing surgery for thoracolumbar IVDD, with improved functional recovery demonstrated in a blinded RCT [16]. In contrast, evidence supporting epidural MPA for DLSS and for perineural or paravertebral administration is derived primarily from retrospective studies and small case series, which limits confidence in treatment efficacy [13–15]. Likewise, evidence for musculoskeletal applications is largely confined to retrospective clinical management studies rather than controlled therapeutic trials [20]. Contemporary evidence supporting routine systemic IM depot MPA as an evidence-based treatment for naturally occurring canine diseases remains sparse, and no head-to-head studies comparing depot MPA with short-acting oral glucocorticoids were identified.

### Interpretation of the current evidence base

The limited number of controlled clinical efficacy studies should not be interpreted as evidence that depot MPA is a newer, more potent, or inherently more effective glucocorticoid. Rather, the scarcity of clinical trials likely reflects important methodological and practical challenges in evaluating depot formulations. Because depot MPA cannot be readily withdrawn after administration, rescue treatment protocols are more difficult to implement, and ethical considerations may complicate study design and informed consent. Placebo-controlled trials are also challenging in painful or immune-mediated diseases, whereas heterogeneity in case definitions, treatment protocols, and outcome measures reduces trial efficiency. Furthermore, the long-standing off-label use of depot MPA may have diminished commercial incentives to conduct large comparative clinical trials. Consequently, the available literature is weighted toward PK and physiologic investigations demonstrating prolonged biologic activity rather than toward disease-specific comparative effectiveness studies. It should also be recognized that many of these physiologic and PK investigations were conducted several decades ago using historical formulations and analytical techniques that may not fully represent current manufacturing standards, assay sensitivity, or contemporary clinical dosing practices.

### Potential sources of bias and limitations of the evidence

Potential publication bias should also be considered when interpreting the available evidence. Early physiologic investigations published during the 1980s primarily focused on measurable endocrine and clinic-pathologic changes, including adrenal suppression following depot MPA administration [2–4]. Consequently, these studies may preferentially report detectable physiologic effects while providing limited information regarding clinically meaningful efficacy outcomes. Moreover, differences in formulations, dosing strategies, and outcome assessment methods between historical and contemporary studies further limit direct comparisons across the evidence base.

The generalizability of the highest-quality clinical evidence also warrants careful consideration. The blinded RCT by Natsios *et al.* [16] evaluated a specific local intraoperative epidural application performed during decompressive surgery for thoracolumbar IVDD in a hospital setting. Therefore, the demonstrated improvement in postoperative functional recovery should not be extrapolated to nonsurgical patients, outpatient management, repeated depot injections, or systemic IM administration, where patient populations, timing of treatment, disease severity, and concurrent interventions differ substantially.

### Contextual evidence from related glucocorticoid studies

Evidence from the broader depot glucocorticoid literature may provide additional context regarding the overall benefit-risk profile. Comparative investigations involving intra-articular corticosteroids in canine OA [17] and recent studies demonstrating prolonged recovery of the HPA axis following glucocorticoid exposure [19] support the concept of sustained biologic activity associated with depot corticosteroid formulations. Nevertheless, these findings cannot substitute for well-designed controlled clinical trials specifically evaluating systemic IM depot MPA in naturally occurring canine diseases. Consequently, evidence from related corticosteroid preparations should be interpreted as supportive pharmacologic context rather than direct evidence of clinical efficacy for depot MPA.

### Clinical implications

From a clinical perspective, an important limitation of depot MPA is the inability to titrate the dose, taper treatment, or rapidly discontinue therapy after administration. Unlike oral prednisolone or prednisone, depot formulations cannot be promptly adjusted if undesirable effects develop. Consequently, adverse effects such as PU/PD, polyphagia, opportunistic infections, gastrointestinal complications, excessive immunosuppression, or steroid-induced endocrinopathies may persist until the depot preparation has been completely metabolized. This limitation becomes particularly important when the initial diagnosis is subsequently revised or prolonged glucocorticoid therapy is no longer indicated. Therefore, for common systemic indications, including allergic dermatitis, immune-mediated diseases, and inflammatory gastrointestinal disorders, short-acting oral glucocorticoids continue to offer substantially greater therapeutic flexibility and a more favorable benefit-risk profile in routine veterinary practice.

### Future research directions

Future investigations should prioritize adequately powered RCTs directly comparing depot MPA with short-acting oral glucocorticoids for naturally occurring canine diseases. Standardized diagnostic criteria, validated patient-centered outcome measures, consistent AE reporting, and long-term follow-up are needed to better define the benefit-risk profile of depot MPA. Based on the current evidence, future research is likely to be most clinically valuable for selected localized or procedural applications, such as epidural administration for specific neurologic conditions, rather than for routine systemic IM administration across a broad range of inflammatory diseases.

## CONCLUSION

This CAT demonstrates that the clinical evidence supporting MPA in dogs is highly route- and indication-specific rather than broadly applicable across inflammatory diseases. The strongest evidence was identified for intraoperative epidural MPA administered during decompressive surgery for thoracolumbar IVDD, where a blinded RCT demonstrated significantly faster postoperative functional recovery compared with controls [16]. In contrast, evidence supporting epidural administration for DLSS was limited to prospective and retrospective clinical studies with methodological limitations and high relapse rates, while evidence for perineural, musculoskeletal, and other localized applications was restricted to small case series or retrospective investigations. Systemic IM depot MPA was supported predominantly by PK, endocrine, and physiologic studies demonstrating prolonged systemic exposure and sustained HPA axis suppression, but not by robust disease-specific efficacy trials in naturally occurring canine diseases.

A major strength of this CAT is the comprehensive synthesis of evidence across multiple routes of administration, integrating clinical efficacy studies with PK, physiological, and safety investigations to provide a balanced assessment of the overall benefit-risk profile of MPA in dogs. The use of a predefined PICO framework, systematic literature search, formal quality appraisal using design-specific risk of bias tools, and emphasis on patient-important clinical outcomes further strengthen the reliability and clinical relevance of the evidence synthesis.

Nevertheless, the available evidence has several important limitations. Most studies involved small sample sizes, heterogeneous study populations, variable outcome measures, inconsistent AE reporting, and limited long-term follow-up. Furthermore, only one blinded RCT evaluated clinically meaningful efficacy in naturally occurring disease, while many physiologic studies were conducted several decades ago using historical formulations and analytical methodologies. The absence of direct comparative studies between depot MPA and short-acting oral glucocorticoids further limits evidence-based therapeutic decision-making.

Overall, current evidence supports MPA as a pharmacologically long-acting glucocorticoid with proven biologic activity in dogs. However, evidence supporting disease-specific clinical efficacy remains limited and largely confined to local applications, particularly intraoperative epidural administration for thoracolumbar IVDD. Until stronger comparative evidence becomes available, routine systemic IM depot MPA cannot be considered an evidence-based alternative to short-acting oral glucocorticoids for most naturally occurring canine diseases, and treatment decisions should remain individualized based on the clinical indication, anticipated benefits, potential risks, and the need for therapeutic flexibility.

## CLINICAL RECOMMENDATIONS

Current evidence indicates that epidural MPA administered intraoperatively during decompressive surgery for thoracolumbar IVDD has moderate-quality evidence supporting faster short-term functional recovery and may therefore be considered an evidence-based option for this specific localized indication. For dogs with DLSS, the available evidence suggests potential short-term clinical benefit following epidural administration. However, the absence of RCTs, frequent relapse during follow-up, and important methodological limitations reduce confidence in the durability and magnitude of the observed treatment effects. Similarly, evidence supporting perineural, paravertebral, or other localized applications remains limited to small observational studies, precluding strong evidence-based recommendations.

For common systemic indications, including allergic dermatitis, immune-mediated diseases, inflammatory bowel disease, and other chronic inflammatory disorders, the evidence supporting IM depot MPA remains of low certainty. Available data are derived predominantly from PK, endocrine, and physiologic investigations demonstrating prolonged systemic exposure and sustained HPA axis suppression rather than from controlled clinical efficacy trials conducted in naturally occurring disease. Consequently, current evidence is insufficient to support routine use of systemic IM depot MPA as a first-line evidence-based treatment for these conditions.

In routine clinical practice, short-acting oral glucocorticoids generally offer greater therapeutic flexibility because treatment can be individualized through dose adjustment, gradual tapering, or prompt discontinuation if adverse effects develop or the diagnosis changes. These characteristics may provide a more favorable benefit-risk profile for many systemic inflammatory and immune-mediated diseases. Nevertheless, depot MPA may remain a reasonable therapeutic option in carefully selected cases where oral administration is impractical, long-term treatment adherence is unlikely, repeated handling would adversely affect patient welfare, or individual clinical circumstances favor prolonged drug exposure. In such situations, clinicians should ensure that owners are fully informed about the prolonged duration of action, the inability to rapidly reverse treatment once administered, and the potential risks associated with sustained glucocorticoid exposure before treatment decisions are made.

## DATA AVAILABILITY

The supplementary data can be made available from the corresponding author upon request.

## GENERATIVE AI DECLARATION

The authors declare that generative artificial intelligence (AI) tools were used solely to improve language, grammar, and readability during manuscript preparation. All scientific content, data analysis, interpretation of results, and conclusions were developed and verified by the authors. The authors take full responsibility for the accuracy, integrity, and originality of the work presented, and no AI tool was listed as an author.

## AUTHORS’ CONTRIBUTIONS

JK: Conceptualization, study design, literature search, study screening, data extraction, risk of bias assessment, evidence synthesis, and writing-original draft preparation. AI: Conceptualization, study design, literature search, study screening, supervision, methodology, critical review, and editing of the manuscript. Both authors read and approved the final version of the manuscript.
